# Whole Grain Muffin Acceptance by Young Adults

**DOI:** 10.3390/foods7060091

**Published:** 2018-06-13

**Authors:** Thomas Mellette, Kathryn Yerxa, Mona Therrien, Mary Ellen Camire

**Affiliations:** 1 WakeMed Health & Hospitals, Raleigh, NC 27610, USA; tommellette@gmail.com; 2 Cooperative Extension, University of Maine, Orono, ME 04469-57417, USA; kate.yerxa@maine.edu; 3 School of Food & Agriculture, University of Maine, Orono, ME 04469-5735, USA; mona.therrien@maine.edu

**Keywords:** whole grains, nutrition knowledge, consumer, baking, sensory evaluation

## Abstract

Adolescents and young adults in the United States do not consume recommended amounts of whole grains. University dining services have opportunities to inform students about whole grains and to offer foods containing blends of whole grains with refined flour to increase daily consumption of these healthful foods. An online survey of university students (*n* = 100) found that 70% of respondents did not know the proportion of servings of whole grains that should be eaten daily. Mini blueberry muffins containing 50, 75, and 100% white whole wheat flour were served to 50 undergraduate students who rated their liking of the muffins using a nine-point hedonic scale. Respondents liked all muffin formulations similarly for appearance, taste, texture and overall liking. After the whole grain content of each muffin was revealed, 66% of students increased their liking of the muffins containing 100% whole wheat flour. Only half of the students increased their liking for the 75% whole wheat flour muffins, and most students reported no change in liking for the muffins made with the lowest percentage of whole wheat flour. Labeling whole grain foods in university foodservice operations may increase consumption of this food group by some students. Further research with actual purchase behavior is needed.

## 1. Introduction

Increased consumption of refined grains and lower consumption of whole grains has been associated with increased risks for developing health problems including obesity, cardiovascular disease, type 2 diabetes, and cancer [1,2,3,4]. Health Canada [5] and the United States Dietary Guidelines for Americans [6] recommend that half of all grain servings be whole grains. Foods made with enriched refined flour can contribute essential nutrients and comprise a significant portion of many Americans’ diets according to the National Health and Nutrition Examination Survey (NHANES) [7]. Whole grain and dietary fiber consumption are low, thus Kranz et al. [8] recommended that public health messages focus on high fiber whole grain foods. Foods consumed at breakfast supplied more than 40% of whole grains consumed by children and adults according to the 2001–2010 NHANES, yet less than 20% of dietary fiber consumption occurred at breakfast in children and adults under the age of 51 years [9]. 

The eating habits of young adults are a concern since avoidance of whole grain foods leads to increased risk of disease later in life, and poor dietary choices may be passed on to the next generation [10]. Consumption of whole grains by 9–12-year-old children was dependent upon the availability of such foods at home [11]. Parents of young children in Northern Ireland became interested in introducing additional whole grain foods to their children after learning about the health benefits associated with these foods [12]. Larson et al. [13] concluded that for both sexes, availability of whole grain bread at home, penchant for the taste of whole grain bread, and self-efficacy to eat three or more servings of whole grains daily were strong (*p* < 0.001) predictors of whole grain consumption and frequency of fast food consumption was an indicator for lower whole grain intakes.

A diet recall study of 202 undergraduate college students found that 86% reported eating whole grains, but approximately 69% of all surveyed students did not consume the recommended three servings per day [14]. Rose et al. [15] reported that among 159 college students with a mean age of 19.9 years, fewer servings were consumed than by any other age group. Based on food records in that study, the college students consumed on average 0.7 servings of whole grains per day, which is 37.5% less than the 1.12 servings reported for all Americans [16]. 

One reason for this low whole grain intake could be due to a misunderstanding by college students of the dietary recommendations for whole grain consumption and what constitutes a whole grain food. A survey among 72 college students 18–23 years of age found that only 3% were able to identify the current Dietary Guidelines for Americans whole grain recommendations [17]. The majority of those survey respondents could not identify whether or not foods were made with whole grains. Undergraduate college students who had taken a nutrition course were more likely to know the current whole grain recommendations and were more likely to associate whole grain consumption with better health outcomes [18]. Magalis et al. [19] studied 69 California college students and reported that the students did not recognize or understand whole grains and over-estimated their whole grain consumption. College students 18–24 years old increased their whole grain consumption from 0.37 to 1.16 servings per day after taking a general nutrition course [20]. There is little information regarding the availability of whole grain foods on college campuses in the U.S. Whole grain intake among college students is partly dependent upon the availability of whole grain foods on campus [15]. An assessment of 15 college campuses across the U.S. between 2009 and 2011 for healthful dining options found that dining halls were more likely to have healthful food options than student union or snack-bar type locations, but the authors concluded that college dining locations overall offer limited healthy options [21]. 

Taste is among the top determinants of food choice for people in the U.S. [22]. Australian university students (*n* = 1306) who rated taste as highly important for food selection had poorer quality diets and were more likely to consume foods made with refined grains such as cakes, pastries, biscuits, and pizza [23]. Adolescents in the United Kingdom were aware that whole grain foods were more healthful than foods made with refined grains, but disliking of the sensory properties of whole grains was the primary obstacle to consumption [24]. For young adults, the association of poor taste with whole grains may create a bias against food products that have their whole grain content labeled prominently on the front of the package. A qualitative study of Northern Ireland adults’ attitudes indicated that serving whole grains “in disguise” might counter the prevalent belief that whole grains have inferior sensory quality [12].

The main objectives of this research project were to understand the reasoning behind college students’ whole grain food choices and whether students would accept muffins made with white whole wheat flour. The primary hypotheses for the online survey were that University of Maine students did not understand whole grain consumption recommendations and that believing whole grain foods were healthful would not influence their interest in consuming more whole grains. The research hypothesis for the acceptability study was that university students would find muffins made with white whole wheat flour to be acceptable and that liking would increase when the whole grain content was displayed.

## 2. Materials and Methods 

### 2.1. Student Sample

The University of Maine is a land grant institution located in the greater Bangor region that has a population of approximately 153,000 people [25]. In the academic year 2014–2015, the university had an enrollment of 9339 undergraduates and 1947 graduate students. Among the undergraduate population, 48% were female, 78% were Caucasian, and 74% were Maine residents [26]. Students were recruited through the campus email conferencing system. Criteria for inclusion in the study were being aged 18–24 years and an undergraduate student at the University of Maine. Students majoring in food science or human nutrition were excluded from the study because they may have more knowledge about whole grains than would other students [18]; nutrition majors were more likely to follow the Dietary Guidelines for Americans to choose grains, fruits, and vegetables than were students in other majors [27]. Students in food science and nutrition represented less than 2% of the total undergraduates at the University of Maine matriculating at the time of the study. Persons with an allergy, intolerance, or aversion to foods containing wheat or dairy were asked to not participate in the sensory evaluation. The University of Maine’s Institutional Review Board (IRB) approved the study protocols on March 7 2014 and judged the research exempt from further review.

### 2.2. Survey Instrument

A 20-question Internet survey was developed with Qualtrics software (v. 60262, Qualtrics LLC, Provo, UT, USA) was pilot-tested with 21 University of Maine faculty and students over the age of 18 years. The questions were designed to assess participants’ access to and knowledge of whole grains and how well they liked whole grain products. Convenience sampling was used to recruit potential participants. When the interested parties selected the email link listed in the recruitment notice, they were brought to a web page showing the informed consent form. Prospective participants were informed of the $5.00 cash compensation for survey participation. Individuals could choose to either begin the survey or exit the window and not participate. At the end of the survey, participants were given the option of entering their e-mail address should they wish to receive further instruction on how to collect their compensation. The survey closed when 100 surveys were completed. 

### 2.3. Sensory Evaluation

A wild blueberry muffin recipe [28] was modified to yield three treatments containing 50%, 75%, and 100% white whole wheat flour (King Arthur, Norwich, VT, USA) in combination with all-purpose flour (Hannaford Bros. Co., Scarborough, ME, USA) (Table 1). The 3 × 4 mini-muffin pans (30 mL volume; Wearever, Lancaster, OH, USA) were placed on a full-size sheet tray (The Vollrath Co., L.L.C., Sheboygan, WI, USA) and coated evenly with pan release spray (Par-Way Tryson Co. St. Clair, MO, USA). Seventeen g of muffin batter was then placed into each of the pan slots with a 22-mL scoop (The Vollrath Co., LLC, Sheboygan, WI, USA). The sheet tray was placed into the oven at 204 °C for a total of 30 min, with pan rotation every 10 min. The muffins were cooled on wire racks for 20 min and were placed into 7.1L plastic storage trays (Carlisle Companies, Inc., Charlotte, NC, USA). Each layer of muffins was separated with deli paper (James River Corp., Parchment, MI, USA) and the container was covered with plastic wrap (Reynolds Consumer Products LLC, Lake Forest, IL, USA) overnight before sensory evaluation.

After participants had read the informed consent form for sensory evaluation, they were escorted to one of 12 partitioned cubicles with climate control and simulated Northern daylight that meets ASTM guidelines for sensory evaluation laboratories [29]. Each cubicle contained a Microsoft^®^ Windows computer. SIMS2000 sensory evaluation software (version 6.0, Sensory Computer Systems LLC, Berkeley Heights, NJ, USA) was used to create the questionnaire, randomize sample order presentation, and collect and analyze data. Demographic questions were asked first, and then participants received a tray containing one muffin for each of the three test formulations. Each formulation was labeled with a randomly-selected three-digit code. Sample order presentation was also randomized. All muffin samples were evaluated using a nine-point hedonic scale (1 = dislike extremely; 9 = like extremely) for appearance, flavor, texture, and overall liking [30]. Participants were also given spring water (DZA Brands LLC, Salisbury, NC, USA) to drink between each sample. After completing the questions for each randomized sample, the whole grain content and the number of servings of whole grains that a typically-sized muffin would provide were revealed to each participant. Participants were then asked whether overall liking for the sample was changed, if at all, by learning the amount of whole wheat flour present using a five-point Likert scale (decreased considerably, decreased somewhat, did not change, increased somewhat, increased considerably). Upon completion of the test, participants were given $10 cash compensation.

### 2.4. Color Analysis

Muffins without blueberries were baked for color measurement to assess the color of the muffin batter only since the number and size of berries per muffin could not be controlled. CIE L* a* b* color values were measured with a LabScan XE Spectrophotometer (Hunter Associates Laboratories, Inc., Reston, VA, USA) using Hunter Lab universal software (v. 4.10, Hunter Associates Laboratories, Inc., Reston, VA, USA). A port size of 50.8 mm and area view of 44.5 mm was used. Samples were placed on a watch glass (Corning Inc., Corning, NY, USA). Nine samples of each muffin variety were used to measure L* a* b* values. Muffins were cut in half vertically. The average of three readings for each of the nine samples was used for statistical analysis. 

### 2.5. Statistical Analyses

Descriptive statistics for the survey were completed by the Qualtrics v. 60262 (Provo, UT, USA). Sensory evaluation data were analyzed with SYSTAT 12 software (v. 12.00.08, Systat Software, San Jose, CA, USA). Hedonic scores and changes in liking were compared by analysis of variance and Tukey’s honestly significant difference test with a significance level of *p* ≤ 0.05.

## 3. Results

### 3.1. Survey Results

#### 3.1.1. Survey Respondent Characteristics

One hundred students completed the survey; the respondent gender distribution was 53% male and 46% female; one participant chose not to answer (Table 2). The University’s undergraduate population at the time of the study was 52% male and 48% female [26]. The median age of the study participants and full-time students at the University was 20 years; the median year of school for survey respondents was second. Most respondents (53%) stated they lived on campus or in a sorority/fraternity building. The median age of those respondents who lived on campus was 19 years, and for those who lived off campus, 21 years. The number of participants on a college dining plan was split with 47% responding no and 47% responding yes, for most meals. Of those students who lived off campus, 80% reported that they somewhat or very much felt they had better access to whole grains than they would if they lived on campus, compared with 41% of students who lived on campus who claimed they had better access to whole grains than they would if they lived off campus.

#### 3.1.2. Food Habits of Survey Respondents

While daily breakfast was not a habit for 60% of respondents, the median days per week breakfast was eaten was six days. Twenty-seven students reported eating breakfast four or fewer days per week. Eighteen-year-olds were less likely to eat breakfast than were 20, 21, and 22-year-olds (*p* ≤ 0.05). Students in their first year of university were less likely to eat breakfast than were third and fourth-year students (*p* ≤ 0.05). There were no significant differences between gender and likelihood to eat breakfast. Those who lived on campus were less likely to eat breakfast than those who lived off campus (*p* ≤ 0.01).

Participants were asked to rank four factors in order of importance when selecting a meal (data not shown). Ninety-three people answered this question. Taste was most often selected as being most important (*n* = 31), followed by health and cost with the same number of responses (*n* = 28), and convenience had the least amount of responses (*n* = 6). However, when the rank sums of all responses were compared using the Wilcoxon signed rank test, cost and taste were ranked equally important, health was less important than cost, convenience was ranked as least important. 

To assess whether a ‘health food’ assumption existed towards whole grains, we asked participants if they considered whole grains to be a ‘health food’ (Table 3). All of the respondents answered this question, and 63 answered ‘yes’ while 25 said ‘no’ and the remaining 12 were ‘not sure’. When asked how they agreed with the statement “health foods usually taste bad”, 70% stated they either disagreed or strongly disagreed. Thirty-five percent of respondents reported that they either sometimes or always feel in general that whole grain foods would not taste good. 

Respondents were asked to self-report their understanding of what whole grain means. Of those who answered (*n* = 99), 69% stated they felt that they had a good understanding of what whole grain means, and 31% either selected no or not sure. When asked what percentage of their total grain intake should be whole grain, 70% of respondents did not reply with the correct answer of 50%. In response to the question “do you in general prefer whole grain foods over non-whole grain foods?”, 46% had no preference or answered no. Fifty-four percent reported that they prefer whole grain foods over non-whole grain foods. Females were more likely to prefer whole grain foods than males (*p* ≤ 0.01). “Too expensive” was the top reason for not eating whole grains, followed by “it won’t taste good”. 

Nearly 45% of survey respondents were either neutral to or not likely to purchase a packaged food with the words ‘whole grain’ clearly labeled on the front, and only 22% were very likely to buy such products. Females were more likely than males to purchase a packaged food with the words “whole grain” clearly labeled (*p* ≤ 0.01). The gluten-free diet has become increasingly popular in American society both for those who require it as medical nutrition therapy and for those who avoid gluten by choice. Fourteen participants reported that they at least occasionally avoided eating foods that contain gluten, and all of these students avoided gluten-containing foods due to personal choice only. One respondent did state he/she ate less whole grains because of omitting gluten, two reported eating more whole grains, and 11 reported there is no difference in whole grain intake.

### 3.2. Consumer Evaluation of Whole Grain Muffins

#### 3.2.1. Participant Demographic Traits

The median age of the 50 participants was 20 years, and the median year of university was the third year. The gender distribution was split evenly; two persons chose to not answer the question. Although 44% of participants lived on campus or in a fraternity/sorority building, the majority of students were responsible for procuring their own food off-campus. Sixty-eight percent reported having a good understanding of what whole grain means, but 78% answered incorrectly when asked which percentage of total grain intake is recommended to be whole grain.

#### 3.2.2. Hedonic Assessments

**Figure 1:**
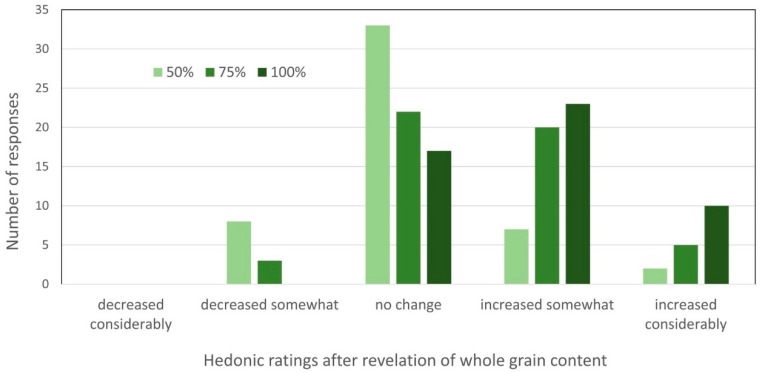
Change in overall acceptance of muffins after the revelation of whole grain content ^a^. ^a^ Muffins contained 50–100% whole wheat flour.

Mini-muffins were prepared so that a whole muffin could be served without creating sensory fatigue. The mean scores for the three samples of mini-muffins were between 6.5 and 7.2 (like slightly to like moderately) but no significant differences were found (Table 4). After each sample was evaluated, the percentage of white whole wheat flour used in each sample was revealed, and participants were asked to rate how their overall liking of the sample had changed, if at all, using a five-point scale: (1 = “decreased considerably,” 3 = “no change,” and 5 = “increased considerably”). The increases seen in overall liking of the 100% and 75% white whole wheat mini-muffins were significantly higher than those for the 50% whole wheat sample, and the 100% was re-rated higher than was the 75% whole wheat sample (*p* ≤ 0.05) (Figure 1). 

### 3.3. Color 

Since it is difficult to control the amount of blueberries per muffin and any associated anthocyanin bleeding, muffins were prepared without blueberries solely for crumb color analysis. The exterior CIE L* and b* of the muffins did not vary among the treatments; CIE a* was significantly (*p* ≤ 0.001) less red for the 50% whole muffins. Although the interior crumb color was significantly different between the 100% whole wheat and the other muffin types (Table 5), the differences were relatively small.

## 4. Discussion

### 4.1. Survey Findings

The demographics of this study’s participants were representative of the University’s undergraduate population at the time of the study. Human nutrition/dietetics and food science students were excluded from the study because persons with nutritional education are more likely to know about whole grains and are thus more likely consume whole grain products [20,27]. The median age and year of school were very similar to other comparable whole grain studies involving college students [14,31]. Two- and four-year college students in Minneapolis (*n* = 1013) reported eating breakfast only 4.2 days per week [32]. Approximately 25% of participants in our survey reported eating breakfast four or fewer days per week. These are critical findings because Americans eat the majority of their whole grain intake during breakfast [16]. The present survey’s findings suggested that students became more inclined to eat breakfast during their later college years, but this study lacked statistical power to support this inference. Students who lived off campus (80%) felt that they had better access to whole grain foods than they would if they lived on campus. Thirty-five percent of those who lived on campus felt that they would have better access to whole grains if they lived off campus. While healthful options may be limited at colleges across the country [21], data were not found on the availability of whole grain options in campus dining facilities. Increased availability of whole grains in restaurants as well as homes has been advocated to increase whole grain consumption by adolescents and young adults [13]. University dining services often provide multiple meals per day to undergraduate students and therefore present an opportunity to expose students to these foods.

When survey participants ranked their top four reasons for food choices, taste was most often selected as being number one, which is consistent with other research in the United States [22,33]. Whole grains are perceived to cost more than refined grain products [12]. The perceived healthfulness of whole grains and the notion that ‘health foods’ are likely to have poor sensory characteristics were explored, and findings were consistent with previous research that studied the reasons why people choose not to eat whole grains. One study showed that of those who reported they did not eat whole grain products, 45% indicated this was because they disliked the taste or texture [14]. A focus group study among adults found that many of the participants disliked whole grain products because of their sensory characteristics [12]. While taste is certainly relative to the individual, this may indicate that further education is needed to demonstrate that whole grain foods can be prepared in a variety of favorable methods. 

To measure knowledge of whole grains and whole grain recommendations, participants were asked to self-report their perceived knowledge level. However, less than a third of respondents in this study who answered that they had a good understanding could correctly identify which percentage of daily grains should be whole grains. This finding indicates the need for nutrition education among these individuals. In a study comparing college students who have had a nutrition course and those who have not, those who had taken a nutrition course were both more likely to know whole grain recommendations and more likely to correctly identify whole grain products [14]. Given the increasing prevalence of chronic diseases that can be prevented with consumption of whole grains, a general nutrition course should be encouraged for all college majors. At the University of Maine, for example, the introductory food and nutrition class satisfies the general education requirement for applications of scientific knowledge.

Females were more likely to prefer whole grain foods and were also more likely to purchase a packaged food with the words ‘whole grain’ clearly labeled on the front of the package (*p* ≤ 0.05). Nearly half (*n* = 45) of survey respondents were either neutral or not likely to purchase a packaged food with the words ‘whole grain’ clearly labeled on the front. This result further strengthens the hypothesis that for those with a bias against whole grain foods, whole grain labeling may deter students from purchasing those foods. However, for those who do prefer whole grain products, clear package labeling appears to be helpful for whole grain identification. People who seek out whole grain products often rely on food packaging or advertisements for guidance [18,20]. Therefore, it may be difficult to market a single whole grain product to both those who prefer whole grains and those who do not. 

To overcome this challenge, the substitution of white whole wheat flour for regular all-purpose flour in various foods may be advantageous along with not labeling whole grain content on the front of the package. The students who prefer whole grain foods may read the ingredients list and identify that the product is made with whole grains and those who do not prefer whole grains may find sensory characteristics of the product appealing. White whole wheat tends to have a mild flavor, smooth texture, and a light color that is aesthetically pleasing to many consumers [34]. The sensory evaluation component of this study tested the hypothesis that a product made with white whole wheat flour that does not have its whole grain content clearly labeled is acceptable to college students. Undergraduate students rated white whole wheat muffins as more healthy (*p* ≤ 0.05) after researchers revealed that the muffins contained whole wheat [35]. 

Among the various reported reasons for not eating whole grain foods, “too expensive” was the top response, followed by “it won’t taste good” and “too hard to prepare”. Taste, expense, and convenience were the top three barriers to whole grain consumption in another study [22]. Among 545 grain-based products evaluated, those containing the whole grain stamp typically cost four cents more per serving of the food [36]. It was not clear how much of the higher cost could be attributed to the fee charged to companies by the Whole Grains Council for the right to display the stamp.

Gluten-free diets have become increasingly popular, but people following such a diet may not have a medical condition that requires avoidance of gluten [37]. Participants in the nurses’ health study and the health professionals’ follow-up study who avoided gluten were more likely to have lower consumption of whole grains, and thus higher risk for cardiovascular disease [38]. Fourteen of the survey respondents in this study reported sometimes avoiding eating foods that contain gluten, and all of these students reported they avoided gluten by personal choice, not due to a confirmed medical diagnosis of celiac disease or gluten intolerance. There may be an opportunity for qualified health professionals such as registered dietitian nutritionists (RDN) to use the preferred media of television and the internet along with in-person education opportunities to distribute evidence-based nutrition information to college students. Education about whole grain gluten-free foods may also be needed for persons with celiac disease. In 2006, Cousineau et al. [39] reported that a web-based personalized nutrition curriculum might be helpful in distributing nutrition information to college students. The growth in popularity of smartphones and other technologies suggest that there may be more opportunities to promote healthful behaviors, especially for students with an internal and powerful other locus of control [40]. 

### 4.2. Sensory Evaluation

Muffins are a popular breakfast food and thus were selected as the test food in this study despite the added sugar in the formulation. A U.S. standard-sized muffin weighs 139 g [41]. Standard muffins made according to the formulations that we used would provide 18, 27, and 36 g of whole grain for the 50, 75, and 100% whole wheat flour formulations, respectively. No significant differences in hedonic ratings were found for any of the tested attributes (appearance, flavor, texture, overall liking). Muffins made with only whole wheat were liked as well as those made with mixtures of refined and whole wheat flours. Even when whole grain content was not prominently labeled, the college students in this study found the product acceptable. However, this study reports on a small sample (*n* = 50) at a small university in a racially-homogenous state. Neo and Brownlee [42] assessed liking of some whole grain foods among young adults in Singapore aged 21–26 years old. A two-week familiarization period increased acceptance of whole grain cookies, granola bars, muesli, and whole grain pasta. The Willingness to Eat Whole Grains Questionnaire has been recently evaluated with university students aged 18–29 years and could be a useful tool in future nutrition education and intervention projects [43].

### 4.3. Strengths and Limitations

The survey yielded information to better inform university dining services about students interests and potential barriers to acceptance of whole grain foods. Limitations of the survey included incomplete representation of the student population and lack of self-reported whole grain consumption data and assessment of students’ ability to recognize whole grain foods. The whole grain muffin sensory evaluation provided interesting information about university students’ willingness to like the muffins containing a greater percentage of whole wheat flour, but we cannot rule out the possibility that students sought to please the researchers. Drawbacks of the experimental design were the lack of a control sample containing no whole wheat, and the inclusion of only white whole wheat flour. Comparisons of products made with refined flour, white whole wheat, and red whole wheat are needed. Baking conditions were optimized for muffins containing whole wheat, thus potentially causing an inferior muffin containing only refined flour. Future work should include bread, wraps, and other non-sweet types of grain products, and involve students from several universities since the University of Maine is a relatively small institution without a diverse student population. Attitudes towards and knowledge of whole grains by young adults who are not pursuing university degrees are other important topics that should be considered for future research.

Nutrition behavior research on healthful food selection has focused on children since interventions at an early age are expected to lead to lifelong habits. A systematic review of children’s food choices identified four important learning processes: familiarization, observational learning, associative learning, and categorization of foods [44]. A greater understanding of the processes used by young adults to select foods as they transition to independent living is needed. Familiarization with whole grain foods was evaluated in a group of overweight adults who consumed less than 20 g of whole grains daily [45]. The intervention groups were provided with whole grain foods and asked to consume three to six servings per day for 16 weeks. Food frequency questionnaires collected over the next 12 months indicated that persons in the intervention groups consumed more whole grains than did the control group members immediately after the intervention period ended, but whole grain consumption declined with time and never approached the 60–120 g levels advocated during the intervention period. Universities have the opportunity to expose students to whole grain foods for periods of approximately eight months per year, thus longer interventions with university students should be conducted. 

## 5. Conclusions

Universities have the opportunity to educate students about whole grain foods and to develop healthful eating patterns that include whole grains. The inclusion of more whole grain foods at breakfast in university dining halls may reach those students who do eat breakfast, but younger students are more likely to skip this meal. Identification of whole grains at local restaurants and markets might assist students as they begin expanding their food options and preparing their meals when they move off-campus. Gender-specific nutrition education messages might be effective [29,46]. Establishment of regular whole grain consumption patterns in young adults could lead to increased popularity of these healthful foods in subsequent generations. White wheat offers foodservice operations and food processors an option to make whole grains more appealing to young adults, particularly those enrolled in a university.

## Figures and Tables

**Table 1:** Muffin formulations (g) ^a,b^.

Title 1	Manufacturer	% White Whole Wheat Flour		
		50%	75%	100%
white whole wheat flour	King Arthur Flour Co. Inc., Norwich, VT	235	352.5	470
all-purpose flour	Hannaford Bros. Co., Scarborough, ME	235	117.5	0
buttermilk	H.P. Hood LLC, Lynnfield, MA	625	625	625
light brown sugar	Hannaford Bros. Co., Scarborough, ME	355	355	355
frozen wild blueberries	Jasper Wyman & Son, Milbridge, ME	215	215	215
vegetable oil	Hannaford Bros. Co., Scarborough, ME	110	110	110
baking powder	Clabber Girl Corp., Terre Haute, IN	12.5	12.5	12.5
iodized salt	Morton, Chicago, IL	10	10	10
vanilla extract	ACH Food Co., San Francisco, CA	10	10	10
Sugar in the Raw^®^	Cumberland Packing Corp., Brooklyn, NY	10	10	10
baking soda	Hannaford Bros. Co., Scarborough, ME	5	5	5
cinnamon	McCormick Corp., Sparks, MD	5	5	5

^a^ Recipe modified from King Arthur Flour Company, Inc. [21]; ^b^ total batter weight: 1827.5 g, producing 107 servings of 17 g muffins.

**Table 2:** Demographic characteristics of University of Maine survey respondents.

Demographic Characteristic	Variable	Number of Respondents	Total Respondents
age (years)	18	14	90
	19	26	
	20	20	
	21	14	
	22	13	
	23	3	
gender	Female	46	99
	Male	53	
years in college	1	28	100
	2	29	
	3	25	
	4	14	
	5 or more	4	
housing situation	On campus or in a fraternity or sorority	53	100
	Off-campus	47	
dining plan	Yes	47	100
	No	47	
	Some meals	6	
weekly breakfast frequency	0–1	4	98
	2–3	23	
	4–6	32	
	7	39	

**Table 3:** University students’ knowledge of and attitudes about whole grains.

Question	Variable	Number of Responses
Does whole grain on the label affect your purchase decision?	Not likely	10
	Neutral	35
	Likely	55
Do you in general feel that products made with whole grains will not taste good?	No	60
	Not Sure	4
	Sometimes	28
	Yes	7
Are whole grains a health food?	No	25
	Not Sure	12
	Yes	63
Do you prefer whole grains?	No	16
	No preference	30
	Yes	54
Reason for not choosing whole grain foods	Won’t taste good	12
	Too expensive	38
	Too hard to prepare	7
	I always try to eat whole grains	26
	Does not apply to me	11
Avoiding gluten?	No	83
	Not Sure	3
	Sometimes	9
	Yes	5
Does avoiding gluten reduce your whole grain consumption?	No	2
	No Difference	11
	Yes	1

**Table 4:** Hedonic scores for blueberry muffins containing different percentages of white whole wheat flour ^a,b^.

Attribute	Percentage White Whole Wheat Flour		
	50%	75%	100%
appearance	7.1 ± 1.3	7.1 ± 1.6	7.0 ± 1.2
flavor	7.2 ± 1.4	7.1 ± 1.3	6.9 ± 1.5
texture	6.9 ± 1.4	6.7 ± 1.5	6.5 ± 1.6
overall liking	7.1 ± 1.3	7.1 ± 1.3	6.7 ± 1.5

^a^*n* = 50; *p* > 0.05, Tukey’s honestly significant difference test. ^b^ 1 = dislike extremely; 5 = neither like nor dislike; 9 = like extremely. *n* = 50. Standard deviations are shown in parentheses next to means.

**Table 5:** CIE interior color of muffins containing different percentages of white whole wheat flour ^A,B^.

Whole Wheat Flour (%)	L*	a*	b*
50	50.4 ± 1.3 a	8.9 ± 0.2 c	27.4 ± 0.8 b
75	48.1 ± 1.1 b	9.3 ± 0.2 b	27.4 ± 0.2 b
100	46.5 ± 1.5 c	9.8 ± 0.2 a	28.1 ± 0.5 a

^A^ Means ± standard deviations (*n* = 9). Color scales: L*, 0 = black, 100 = white; a*, +a = red, −a = green; b*, +b = yellow, −b = blue. ^B^ Values with the same letter in columns are not significantly different (*p* > 0.05).
